# Differential apoptotic gene expression in the male partners of infertile couples with normal and abnormal sperm parameters

**DOI:** 10.5935/1518-0557.20220004

**Published:** 2022

**Authors:** Maliheh Afsari, Ali Reza Talebi, Fatemeh Dehghani Tafti, Masoud Makki, Farzaneh Fesahat

**Affiliations:** 1 Reproductive Immunology Research Center, Shahid Sadoughi University of Medical Sciences, Yazd, Iran; 2 Department of Biology & Anatomical Sciences, Shahid Sadoughi University of Medical Sciences, Yazd, Iran; 3 Parseh Laboratory of Medical Science, Yazd, Iran

**Keywords:** sperm parameters, apoptosis, gene expression, unexplained male infertility

## Abstract

**Objective:**

The purpose of this study was to investigate the cellular and molecular levels of apoptosis induction in three groups of male partners of infertile couples, one featuring subjects with normal sperm parameters and unexplained male infertility (UMI), one including men with abnormal sperm parameters, and one with fertile men as controls.

**Methods:**

Twenty-five infertile men with abnormal sperm parameters and 25 men with UMI and normal sperm parameters were recruited as experimental group I and experimental group II; 25 fertile men were included as controls. The mRNA levels of *Fas, Fas ligand, Caspase 8, Bax*, and *Bcl2* were measured in the three groups. The cellular rates of early and late apoptosis were assessed using annexin V and propidium iodide staining.

**Results:**

The expression of *Bax, Bcl2*, and the *Bax/Bcl2* ratio in experimental group I was significantly higher than that in experimental group II and controls. However, the *Bax/Bcl2* ratio was less than 1 among all groups. No significant difference was found among study groups regarding the gene expression of *Fas, Fas ligand*, and *Caspase 8*. No significant difference was seen in early apoptotic rates of sperm among study groups. The highest number of necrotic sperm cells was detected in experimental group I.

**Conclusions:**

The findings showed that the external pathways of apoptosis were not activated in the absence of external stimuli of sperm apoptosis in ejaculated sperm. Regardless of fertility status, apoptosis gene induction in the internal pathway was associated with abnormalities in sperm motility and/or morphology in men with abnormal parameters.

## INTRODUCTION

Infertility is a major problem in society today, with 15% of couples trying to have a child failing to conceive (Agarwal *et al.*, 2015). Semen analysis is usually the first step in assessing the fertility status of men; however, this method does not provide enough information about the genomic integrity of the male gamete. Abnormal semen parameters are considered an indicator of subfertility in men. However, up to 30% of men with normal semen parameters (normozoospermic) are diagnosed with UMI, since the reason for infertility is unknown (Panner Selvam *et al*., 2019).

Three main theories have been proposed to describe the causes of damage to the sperm nucleus, including impaired replacement of histone by protamine, reactive oxygen species (ROS), and apoptosis (Carrell *et al.*, 2007; Talebi *et al*., 2016). These interlinked molecular events can lead to different clinical and laboratory manifestations in infertile males. Clarifying the nature of sperm defects will contribute to the selection of proper assisted reproduction technology (ART) methods, consequently enhancing ART success rates and ensuring improved offspring health (Agarwal & Said, 2005).

Apoptosis, recognized as a type of programmed cell death, is associated with changes in the morphological and biochemical characteristics of cells. Apoptosis plays a prominent role in several physiological and pathological processes (Majtnerová & Roušar, 2018). According to Erkkilä *et al.* (1997), such programmed cell death is a normal and hormonally controlled phenomenon in the adult human testes. Caspase activation, externalization of phosphatidylserine (PS), changing of mitochondrial membrane potential, and sperm DNA fragmentation (SDF) are recognized as markers of apoptosis in ejaculated human spermatozoa (Shukla *et al.*, 2012). So far, two pathways have been considered for apoptosis, namely external and internal. In the external pathway, death signals are transmitted through transmembrane receptors, while in the internal pathway death signals are sent to the mitochondria. A set of genes, such as *Caspase8, Fas, FasL*, and the *Bcl-2* protein family, are involved in both external and internal pathways (Kiraz *et al.*, 2016).

Since there is no report on the possible involvement of apoptosis in ejaculated human spermatozoa with normal and abnormal parameters in male partners of infertile couples with UMI, this study was designed to compare the cellular and molecular levels of apoptosis induction markers in male partners of infertile couples with abnormal sperm parameters, normal sperm parameters with UMI, and fertile men as controls.

## MATERIALS AND METHODS

### Study population

This cross-sectional study included couples who were referred to obstetrics and gynecology clinic, Yazd, Iran. The male subjects of infertile couples were divided into two groups, one with individuals with abnormal sperm parameters (experimental group I, n=25) and another with men with UMI and normal sperm parameters (experimental group II, n=25). For the control group, 25 fertile men with normal sperm parameters were enrolled. The patients were randomly selected into each experimental group by using a simple randomization method. All procedures were conducted with the approval of the institution’s Ethics Committee. The participants gave written consent before joining the study. After examination and sperm analysis ordered by the treating physician, semen samples were collected from the participants.

The inclusion criteria for controls were as follows: having normal sperm parameters and at least one child aged less than two years. The subjects in both experimental groups had primary infertility. Based on the World Health Organization (WHO, 2010), sperm parameters were defined as normal in this study given that the volume of semen was ≥1.5 mL, the concentration of sperm was ≥15 million/mL, total sperm motility was ≥40%, and normal sperm morphology was ≥4% (WHO, 2010). All participants were aged less than 40 years and had a body mass index below 30, no varicocele disease, no history of smoking or drug use, no infectious diseases or diabetes, and no history of alcohol abuse. Patients with pyospermia (i.e. more than one million WBC per ml of sperm), varicocele, azoospermia, fever, and infectious disease during the last 90 days as well as subjects with genetic problems, reproductive tract infections, inflammatory disease of the reproductive tract, sexually transmitted disease, or erectile dysfunction were excluded from the study.

### RNA extraction and cDNA synthesis

RNA extraction was performed from washed semen samples collected from both experimental and control groups using a total RNA extraction kit (Parstous biotechnology, Iran). The integrity of extracted RNA was assessed by agarose electrophoresis. In addition, the final RNA concentration was assessed based on measurements of absorbance at 260 nm (PhotoBiometer, Eppendorf, Germany). In first-strand cDNA synthesis, 500ng of total RNA with the Revert Aid First Strand cDNA Synthesis Kit was used based on the manufacturer’s protocol (Parstous biotechnology, Iran). The cDNA product was kept at-20°C until use.

### Gene expression assessment

The relative gene expression level was studied based on quantitative real-time polymerase chain reaction (qRT-PCR). Master Mix Green with high ROX™ (Amplicon) together with the StepOne system was utilized in each PCR reaction (Applied Biosystems, CA, USA). For each reaction, cDNA (2 µL), forward primer (1 µL), reverse primer (1 µL), master mix (10 µL), and 6 µL nuclease-free water was set to a total of 20 µL. All the reactions were performed in duplicates. The qRT-PCR protocol was as follows: (10 min at 95°C), followed by 40 cycles of amplification stage at 95°C for 15 s, 60°C for 30 s, and 72°C for 30 s. Following the cycling stage, a melting curve stage was run (Sadeghian-Nodoushan *et al*., 2016). Glyceraldehyde 3-phosphate dehydrogenase (GAPDH) and beta-2-microglobulin (B2M) were utilized as the two reference genes in this study. Table 1 summarizes the oligonucleotide primers employed for all genes. To analyze the relative expression level of each gene, a 2-∆∆Ct technique was used. The mean CT of the two reference genes was also calculated for gene expression analysis.

**Table 1:** Oligonucleotide primers.

Gene	Primer sequence (5’-3’)	Sequence amplified	Product size
*Bax*	Forward- TCAGGATGCGTCCACCAAGAAG Reverse- TGTGTCCACGGCGGCAATCATC	NM_138764.5	103 bp
*Caspase 8*	Forward- ATTTGCCTGTATGCCCGAGC Reverse- CCTGAGTGAGTCTGATCCACA	NM_001351594.2	105 bp
*Bcl2*	Forward- ATCGCCCTGTGGATGACTGAGT Reverse- GCCAGGAGAAATCAAACAGAGGC	NM_000633.3	127 bp
*FAS*	Forward- TGAAGGACATGGCTTAGAAGTG Reverse- GGTGCAAGGGTCACAGTGTT	NM_152872.4	118 bp
*FAS-L*	Forward- GCAGCCCTTCAATTACCCAT Reverse- CAGAGGTTGGACAGGGAAGAA	NM_001302746.2	101 bp
*GAPDH*	Forward- AAATCAAGTGGGGCGATGCTG Reverse- GCAGAGATGATGACCCTTTTG	NM_001256799.3	118 bp
*B2M*	Forward -AGATGAGTATGCCTGCCGTG Reverse -TGCGGCATCTTCAAACCTC	NM_004048.2	106 bp

*GAPDH: Glyceraldehyde 3-phosphate dehydrogenase, B2M; beta-2-microglobulin*

### Annexin V and propidium iodide staining

To investigate the cellular apoptosis induction, double staining was carried out using Annexin V-FITC and *Propidium iodide* (PI) to measure membrane PS exposure according to kit instructions. One of the key indicators of early apoptosis is the exposure of PS on the external side of the plasma membrane. Considering annexin V’s high affinity to PS binding, it was used to identify early apoptotic rate. PI, a red fluorescent intercalating dye, was used as a DNA stain to study dead cells or late apoptotic rate (Hamzeloo-Moghadam *et al*., 2018).

The suspension of 1-5 x 10^5^ cells was centrifuged at 1,500 rpm for 3 min. Then, the supernatant was removed. Next, phosphate-buffered saline (PBS) was added to the cell pellet and centrifuged at 1,500 rpm for 3 min. The supernatant was removed and the cell pellet was re-suspended in 100-500 µl of 1X binding buffer. After that, 10 µl of annexin V - FITC was added to the cell suspension. The cell suspension was then incubated for 15 min at room temperature (RT) and in a dark place. Next, 1-5 µl of the PI solution was added and the cells were incubated for 1 to 5 min at RT and in a dark place. Cell analysis was performed with fluorescence microscopy. The percentage of green sperm cells with early apoptosis (Annexin^+^) and red sperm with final apoptosis (PI^+^ cells) was obtained.

### Statistical analysis

For data analysis, SPSS version 20 (SPSS Inc., Chicago, IL, USA) was used. Data were presented as mean ± SEM. Following data normalization, Student’s t-test and the Kruskal-Wallis test were run to compare gene expression levels between the study groups. Statistically significant values had a *p*<0.05 for both cellular and molecular assessments. The association between apoptosis and protamine deficiency was assessed using Pearson’s and Spearman’s correlation coefficients.

## RESULTS

A total of 75 men were included in the three study groups, which featured infertile patients with abnormal sperm parameters (experimental group I, n=25); infertile patients with normal sperm parameters diagnosed with UMI (experimental group II, n=25); and fertile men with normal sperm parameters (control group, n=25).

### Sperm parameters

As expected, no significant difference was observed between the three study groups in terms of sperm volume (*p*≥0.05). Experimental group I had significantly lower progressive motility in comparison with experimental group II and controls (*p*<0.0001 and 0.0005, respectively). Sperm concentration was significantly lower in experimental group I compared to experimental group II (*p*<0.0002). However, no significant difference was detected between experimental group I and controls regarding sperm concentration (*p*=0.08). In contrast, sperm samples of experimental group I showed significantly higher rates of immotile sperm than the samples from controls and experimental group II (*p*=0.002 and 0.0001, respectively). Non-progressive motility of sperm was significantly higher in experimental group I than in controls (*p*=0.008). Data analysis showed a significantly lower rate of normal sperm morphology in experimental group I than in experimental group II and controls (*p*<0.0001 and *p*<0.0001, respectively). We detected a significant increase in round cells in both experimental groups compared to controls (*p*<0.0001 *vs. p*<0.0001, respectively) (Table 2).

**Table 2:** Comparison of demographic features and sperm parameters between study groups.

Variables	Experimental group I	Experimental group II	Control group	*p*-value
Age (yr)	32.04±3.59	32.42±4.25	30.25±0.92	0.14^a^ 0.13^b^ 0.07^c^
BMI	24.28±3.19	25.08±3.71	22.65±0.32	0.42^a^ 0.41^b^ 0.11^c^
Duration of infertility (yr)	5.75±3.35	6.26±3.67	-	0.82^a^
Volume (ml)	3.73±1.6	3.23±1.46	2.57±0.29	0.58^a^ 0.09^b^ 0.16^c^
Sperm count (×10^6^/ml)	39.71±25.73	75.48±34.35	52.4±33.12	**0.0002^a^** 0.24^b^ 0.08c
Progressive motility	28.69±11.61	41.84±6.787	44.76±8.69	**<0.0001^a^ 0.0005^b^** 0.29^c^
Non-progressive motility	12.13±2.49	10.65±2.85	9.5±2.37	0.07^a^ **0.008^b^** 0.26^c^
Immotile sperm	58.39±12.43	47.4±6.83	43.99±8.66	**0.0001^a^ 0.002^b^** 0.33c
Normal morphology (%)	2.53±0.78	3.94±0.64	4.38±0.58	**<0.0001^a^ <0.0001^b^** 0.86^c^

Data were presented as Mean±SD as well as percentage (%) by using independent Student’s t-test. a: comparison between experimental group I and experimental group II; b: comparison between experimental group I and controls; c: comparison between experimental group II and controls

### Gene expression

No significant change was found in the three study groups regarding *Fas* and *caspase 8* mRNA levels (*p*≥0.05). A significantly higher expression of *Fas ligand* was found in experimental group I compared to controls. The gene expression profile of *Bax* and *Bcl2* led to a significant difference between the experimental group I and experimental group II and controls. The expression level of *Bax, Bcl2*, and the *Bax/Bcl2* ratio was significantly higher in experimental group I than in experimental group II and controls (Table 3).

**Table 3:** Comparison of gene expression profile between three study cases.

Variables	Experimental group I	Experimental group II	Control	*p*-value
***Fas***	1.34±0.35	2.08±0.55	1.7±0.66	0.58^a^ 0.11^b^ 0.44^c^
***Fas ligand***	3.09±1.07	1.74±0.65	1.1±0.46	0.09^a^ **0.02**^b^ 0.39^c^
***Caspase 8***	2.58±0.92	1.91±0.7	1.05±0.37	0.16^a^ 0.05^b^ 0.64^c^
***Bax***	1.85±0.49	0.41±0.18	0.38±0.14	**0.001**^a^ **0.001**^b^ 0.94^c^
***Bcl2***	2.48±0.77	1.17±0.34	0.9±0.39	**0.04**^a^ **0.02**^b^ 0.7^c^
***Bax/Bcl2***	0.71±0.035	0.39±0.029	0.44±0.033	-

Data were presented as Mean±SEM. a: comparison between experimental group I and experimental group II; b: comparison between experimental group I and controls; c: comparison between experimental group II and controls. T-test and Kruskal-Wallis test were applied for statistical analysis.

In addition, data analysis showed a significant direct correlation between *Fas* and *caspase 8* gene expression (r=0.32, *p*=0.01). The strongest correlation was seen between *Fas ligand* and *caspase 8* gene expression (r=0.62, *p*=0.00). Furthermore, a significant positive correlation was observed between *Bax* and *Bcl2* gene expression (r=0.47, *p*=0.00).

### Annexin V and propidium iodide assay

**Figure 1:**
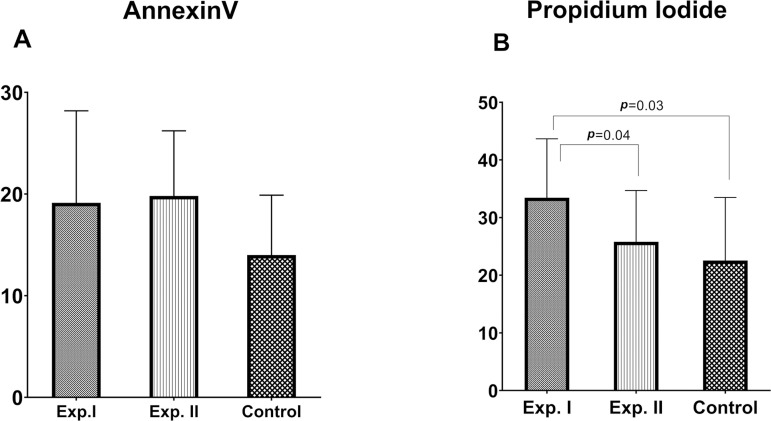
Detection of apoptosis using Annexin V-FITC/PI staining A) Annexin V (+) and B) PI (+) sperm cells indicated early and late apoptosis rates, respectively. Note: Significant differences between the study groups were presented by *p*<0.05.

No significant change was seen in terms of early apoptotic rates (ANXV^+^) of sperm samples between the three study groups. The highest number of necrotic sperm cells (PI^+^) was detected in experimental group I (Figure 1).

There was a significant positive correlation between *Fas* as well as *Fas ligand* mRNA levels and ANXV^+^ sperm (r=0.31, *p*=0.01 *vs*. r=0.4, *p*=0.03, respectively).

## DISCUSSION

The etiology of male infertility seems to be closely related to sperm concentration, motility, and morphology (Wei e*t al*., 2015). Nevertheless, semen analysis is usually not considered a comprehensive method for semen assessment due to its limitations in the evaluation of molecular and cellular levels. On the other hand, sperm DNA integrity is necessary for sperm-egg interactions, fertilization, and early embryonic development (Van Blerkom, 1996).

We detected a significantly higher rates of *Bax* and *Bcl2* gene expression in male partners with abnormal parameters compared to controls. The expression profile of Bcl-2 family members, due to their role in the regulation of apoptotic pathways, is required to assess the survival rate of Sertoli cells, spermatogonia, and spermatocytes. The Bax/Bcl-xl ratio also affects the fate of these cells (Yan *et al*., 2000). Selective expression of Bax and Bcl-2 proteins in germ cells strongly indicates that these proteins are involved in different phases of spermatogenesis, differentiation, and maturation (Oldereid *et al.*, 2001). Bcl-2 proteins can be either pro-apoptotic, such as Bax and Bak, or anti-apoptotic, such as BCL-2, Bcl-xL, and Bcl-w. A fine balance between pro and anti-apoptotic gene function modulates the incidence of apoptosis. For instance, a high ratio of Bax/Bcl-2 reflects a pro-apoptotic tendency (Li *et al*., 2013). Irregular expression patterns of these proteins lead to cell death, fragmentation, and embryo mortality during the early stages of development (Giritharan *et al*., 2007). Dogan *et al*. (2013) demonstrated no significant correlation between male infertility and *Bax* gene expression or PS membrane translocation. They concluded that apoptosis could not be considered as one of the main fertility indicators. As for our findings, it seems that a *Bax/Bcl2* expression ratio of less than 1 might not play a key role in male infertility. Furthermore, given that male partners of infertile couples with UMI showed no significant change in mRNA levels of *bax, blc2*, and *bax/bcl2*, one might conclude that apoptosis induction was more associated with abnormalities in sperm motility and/or morphology.

Caspases play a prominent role in the regulation of apoptosis in the human seminiferous epithelium. Regarding receptor-mediated programmed cell death, caspase 8 has the most important role in death signal transduction (Said *et al*., 2004). According to the results of the annexin V assay, there was no difference between the study groups with respect to the expression of this gene.

We did not find a significant change in *Fas* and *Fas ligand* or caspase *8* in external apoptotic pathways. One of the important apoptosis-related systems in the development of human testicular germ cells is the *Fas* system. Sertoli cells express *FasL*, binding to *Fas* on *Fas*-positive germ cells, and consequently starting apoptosis and restricting the size of the germ cell population to numbers that can be supported. *Fas* ligation induces the trimerization of the *Fas* receptor, activating caspase 8 by an adaptor Fas-associating protein with death domain (Passadaki *et al*., 2013). Since *Fas* upregulation is an external apoptotic trigger, it is possible that the sperm of infertile men have been in a more “hostile” environment than the sperm of fertile men, and that the sperm of infertile men have kept subapoptotic damage (Wang & Su, 2018). Multiple endogenous and exogenous factors are responsible for poor sperm quality and apoptosis, leading to infertility through the production of a surplus of ROS targeted towards healthy spermatozoa. For instance, genital tract infections, varicocele disease, spinal cord damage, diabetes, obesity, smoking, alcohol drinking and recreational drug use, ionizing radiation, psychological stress, freezing and thawing of sperm, strenuous exercise, or air pollutants lead to elevation of ROS (Said *et al.*, 2010; Agarwal & Bui, 2017). Following our exclusion and inclusion criteria, we tried to eliminate the external and/or internal factors affecting ROS production to inhibit the stimulation of apoptosis pathways.

A previous study conducted by our group on a similar population about the impact of abnormal sperm parameters on the mRNA level of some sperm functional genes, oxidative stress, and SDF found significantly higher chromatin anomalies not only in experimental group I with abnormal parameters but also in experimental group II with UMI compared to controls. Moreover, we did not observe a significant change in malondialdehyde levels, a byproduct of oxidation, or total antioxidants among the study groups (Afsari *et al.*, 2021).

Haghpanah *et al*. (2016) studied the impact of SDF on the developmental competence and the incidence of apoptosis after blastomeric biopsy by evaluating both TUNEL and apoptotic gene expression (*BAX* and *bcl2*). They showed that the incidence of apoptosis was not affected by SDF or by blastomeric biopsy. Taheri *et al.* (2015) investigated the association between *Bax* gene expression and the SDF index in sperm cells of infertile men. They showed no significant difference between the two groups with or without SDF in terms of *Bax* expression (Taheri *et al.,* 2015). Sakkas *et al*. (2002) investigated the possible involvement of apoptosis in ejaculated human spermatozoa and found that TUNEL positivity and apoptotic markers were not always present in unison in spermatozoa. Nevertheless, they observed that semen samples with low sperm concentration and poor morphology were more likely to have high levels of TUNEL positivity and *Fas* and *p53* expression. In line with our findings, they found that the presence of DNA damage was not directly related to an apoptotic process happening in spermatozoa and that it was due to problems in the nuclear remodeling process. Accordingly, the existence of apoptotic proteins in ejaculated spermatozoa may be associated with defects in cytoplasmic remodeling during the later stages of spermatogenesis (Sakkas *et al*., 2002).

As expected, the highest number of PI^+^ was found in experimental group I, with a significantly higher frequency of immotile sperm. Similarly, Januskauskas *et al*. (2003) showed a reverse correlation between PI+ sperm cells and sperm motility as well as viability. This finding can be attributed to the fact that PI labels all immotile cells with compromised plasma membranes.

## CONCLUSION

The findings showed that the external pathways of apoptosis were not activated in the absence of external stimuli of sperm apoptosis in ejaculated sperm. Regardless of fertility status, apoptosis gene induction in the internal pathway was associated with abnormalities in sperm motility and/or morphology in men with abnormal parameters.
